# BIM-driven digital twin for demolition waste management of existing residential buildings

**DOI:** 10.1038/s41598-025-13938-9

**Published:** 2025-08-07

**Authors:** Sakdirat Kaewunruen, Yi-Hsuan Lin, Yuxin Guo

**Affiliations:** 1 School of Engineering, University of Birmingham, Edgbaston, B15 2TT UK; 2 Birmingham Centre for Railway Research and Education, University of Birmingham, Edgbaston, B15 2TT UK; 3 School of Engineering and Business School, University of Birmingham, Edgbaston, B15 2TT UK

**Keywords:** Demolition, Waste management, Building information modelling (BIM), Digital twin (DT), Environmental sustainability, Financial-benefit, Recycling, Construction and demolition (C&D), Civil engineering, Mechanical engineering, Environmental impact

## Abstract

With the accelerated development of urbanisation, the construction industry has significantly contributed to environmental degradation due to its substantial energy consumption and construction and demolition (C&D) waste generation. By assessing the ecological impact of the construction industry alongside existing demolition waste management practices, this article aims to develop a conceptual framework to optimise building demolition, transportation, and recycling processes. This study integrates a BIM-driven Digital Twin framework into C&D waste management, aiming to maximise economic benefits and advance the sustainable development of construction practices. Specifically, it simulates the demolition process of an existing townhouse in Washington, D.C., using BIM-Navisworks software and employs a digital twin to update demolition data in real-time. This approach optimises the classification and transportation of demolition waste, enhancing efficiency and sustainability. The study validates the proposed conceptual framework for building demolition waste management through case simulation. Additionally, it utilises BIM-Dynamo software to analyse the economic benefits of demolition waste recycling, demonstrating that a high recycling rate can significantly enhance economic outcomes. The proposed framework leverages BIM technology to optimise demolition and recycling processes, providing a valuable reference for selecting demolition waste management strategies for other buildings.

## Introduction

With the rapid advancement of urbanization, the construction industry has become a primary energy consumer and a significant contributor to environmental degradation^1^.The environmental impact of construction is primarily observed in two key areas: pollution and resource depletion. The pollution results from greenhouse gas emissions, water contamination, and soil degradation, while resource scarcity arises from the inefficient or excessive use of raw materials. To mitigate these environmental challenges, many countries have implemented policies to improve the energy efficiency of buildings and reduce greenhouse gas emissions. However, these efforts have been insufficient in offsetting the overall increase in energy consumption driven by the expansion of construction activities, rising urban population densities, and intensified urban development^2^. In 2018, construction activities alone accounted for 36% of global energy consumption, and when including energy consumption related to the demolition sector, this figure increased to 50% of total global energy use^3^. This highlights the urgent need for more effective strategies to optimise energy usage, reduce waste generation, and promote sustainable resource management in the construction industry.

The construction industry has increasingly advocated for the development of sustainable architecture. However, the direct application of sustainable principles in building design and construction remains limited^4^. This limitation is particularly evident in the continuous rise in the consumption of construction materials, which has led to an annual increase in construction and demolition (C&D) waste generation^5^. Notably, C&D waste has become one of the most significant contributors to global solid waste, surpassing most other categories^6^. Despite its environmental impact, C&D waste holds potential economic value through recycling and resource recovery^7,8^. According to Arogundade et al.^9^, the contractors can implement low-carbon construction practices by actively reducing carbon emissions. However, as Komurlu et al.^10^ noted, the primary challenges hindering the widespread adoption of low-carbon construction are insufficient government support and the high costs associated with construction and operation. These barriers underscore the need for stronger policy frameworks and financial incentives to facilitate the transition toward sustainable building practices.

From a life cycle perspective, recycling construction waste can significantly mitigate environmental impact by reducing raw material extraction and the depletion of natural resources^11^. Proper management and recycling of C&D waste minimises environmental degradation and generates economic benefits, contributing to a more sustainable construction industry^12^. This shift in waste management highlights the importance of considering the end-of-life stage of buildings, encouraging further research into optimising demolition processes to maximise the residual value of materials. Efficient processing of C&D waste is critical in achieving both environmental sustainability and economic viability^13^. With the growing application of Building Information Modelling (BIM) in the construction industry, its role extends beyond construction planning to include demolition management and waste optimisation. For example, Wang et al.^11^ proposed a BIM-based conceptual framework for construction waste management, which enables the estimation of carbon emissions throughout a building’s life cycle. Such advancements underscore the potential of digital tools in enhancing sustainable demolition practices and promoting circular economy principles in the construction sector.

In summary, the construction industry faces significant challenges balancing urbanisation, economic growth, and environmental sustainability. Effectively addressing these challenges requires the integration of advanced digital technologies, particularly BIM and Digital Twin (DT), to enhance the efficiency and sustainability of demolition and C&D waste management processes. However, previous studies have primarily focused on using BIM to estimate demolition rates at the end-of-life stage of buildings, with limited attention given to the detailed treatment pathways of C&D materials, such as reuse, recycling, and landfill disposal, or further the evaluation of financial benefits under different demolition scenarios. To address this gap, the present study contributes by exploring the potential of BIM to support more comprehensive C&D waste management through the integration of virtual models and systematically designed demolition plans. Through this framework, the study aims to facilitate the adoption of more sustainable building practices. This research addresses the following key questions:

- How can BIM and Digital Twin technologies be maximised to optimise building demolition and waste management?
- What factors influence customer’s selection of waste management strategies, and how can these choices be optimised?
- How can C&D waste management strategies be optimised to maximise the economic benefits of recycling while supporting environmental sustainability?

This study explores these issues by proposing an innovative approach to sustainable waste management in the construction industry. The approach will be applied and evaluated through a case study.

## Literature review

### Definition and application of BIM & DT

According to the USA National Building Information Modelling Standards Committee, BIM is defined as a digital representation of a facility’s physical and functional characteristics. It is a shared knowledge resource for information about a facility, forming a reliable basis for decisions during its life cycle from earliest conception to demolition^14^. Unlike traditional Computer-Aided Design (CAD) models, BIM provides a more comprehensive and multidimensional representation of facility information, significantly enhancing multi-party collaboration throughout a project’s lifecycle. While BIM can be developed using CAD models as a foundation, this approach is often inadequate in accurately depicting the real-world conditions of building facilities, as it lacks sufficient detail to represent their actual state^15^.

Furthermore, through an extensive investigation of construction industry professionals, Eadie et al.^16^ demonstrated that BIM can be effectively implemented across the entire construction life cycle, improving project efficiency and promoting environmental sustainability. By integrating BIM into various stages of construction, from planning to demolition, the technology facilitates resource optimization, waste reduction, and enhanced decision-making, reinforcing its role as a key enabler of sustainable construction practices.

DT is widely applicable across various industries and disciplines and serves as a powerful tool for monitoring and optimizing management operations. By collecting real-time data from physical assets, DT technology enables predictive analysis and informed decision-making, ultimately enhancing operational efficiency^17,18^. In the construction industry, DT technology is crucial in extending the capabilities of BIM, facilitating comprehensive management throughout the project life cycle. Its applications include optimizing energy consumption, improving facility management efficiency, and enhancing the overall performance of buildings. Additionally, Radzi et al.^19^ emphasize that Digital Twin technology provides decision-makers with real-time, data-driven insights, reducing reliance on assumptions and leading to more accurate and strategic decision-making. Rausch^20^ further advances the application of DT by improving parameterisation, thereby enhancing the automation of information exchange between physical assets and corresponding virtual models. This strategy significantly contributes to more efficient and accurate decision-making processes. As the construction industry continues to evolve, the integration of intelligent building technologies, such as DT, has become an essential pathway for advancing sustainability, efficiency, and resilience in the built environment^21^.

### Optimising C&D waste management

Practical C&D waste management begins with strategic demolition planning. Yeheyis et al.^22^ assert that the proportion of recyclable materials generated from demolition largely depends on the dismantling method employed. Therefore, selecting the most appropriate demolition method and route is paramount and should be prioritised to maximise resource recovery and mitigate environmental impact. In the early stages of demolition planning, Kim et al.^23^ introduced a BIM-based framework designed to estimate the quantity and composition of dismantled waste during the design phase. This predictive capability facilitates more effective planning, enabling informed decision-making and enhancing overall building lifecycle management. However, Han et al.^24^ highlight that one of the primary barriers to the widespread adoption of BIM in C&D waste management is the difficulty in accurately capturing relevant data and tracking target objects. To address this limitation, the concept of Smart BIM has been introduced, integrating BIM with advanced digital technologies to extend its application scope^25,26^.

Integrating Smart BIM has significantly advanced the application of DT in demolition planning. By incorporating real-time positioning technologies, drones, and smart terminal devices, it becomes possible to dynamically monitor key parameters such as demolition progress, waste generation, and resource allocation, including labour and machinery utilisation. Su et al.^27^ further developed a real-time updating DT model, which dramatically enhances the efficiency of demolition planning by providing continuous, data-driven insights. The transformation of BIM into a DT framework mitigates the limitations of traditional BIM-based demolition planning, allowing for a more adaptive and responsive approach. Boje et al.^28^ regard DT as a vital enhancement to BIM, which transitions from a static data repository to an interactive and interconnected data network, offering real-time and high-precision data for decision-making.

At the waste management level, Yeheyis et al.^22^ proposed a C&D waste management framework based on the building lifecycle, aligning with government policies promoting the reuse, recycling, and reduction (3R) of construction materials while minimising the environmental impact of waste disposal. Building upon this foundation, Kang et al.^29^ introduced a data-driven approach to C&D waste management, incorporating detailed solid waste transportation planning based on on-site and off-site recycling strategies. The proposed framework calculates costs and benefits under varying recycling scenarios, significantly improving waste management efficiency while offering stakeholders, including contractors, more flexible decision-making options. Saeed et al.^30^ proposed a waste management framework designed to manage construction waste during the demolition phase effectively. The findings of their study indicate that implementing efficient C&D management can lead to significant optimisation in energy consumption and the conservation of natural resources (e.g., water) while also contributing to a substantial reduction in carbon emissions.

Expanding on these advancements, Su et al.^27^ developed a comprehensive DT-driven framework for C&D waste trading systems. This framework integrates DT technology to optimise demolition strategies and incorporates real-time C&D waste estimation models. By leveraging internet-based trading platforms, the system streamlines the entire waste management lifecycle, enhancing resource circulation and promoting a more efficient and sustainable approach to C&D waste management. Suh et al.^31^ further advanced the design methodology for building material reuse by developing an optimisation framework inspired by A*, heuristic and genetic algorithms, which accounts for the structural connectivity and compatibility of reclaimed material stocks. This approach enhances the efficiency and feasibility of incorporating reused materials into new construction designs.

### Knowledge gap

BIM and DT technologies offer significant potential for improving demolition waste management. However, several challenges remain in effectively implementing these technologies in practice. While the C&D waste management framework proposed by Yeheyis et al.^22^ encompasses the entire building lifecycle from planning and design to demolition, it primarily focuses on waste treatment and its environmental impact. However, this framework lacks consideration for waste transportation logistics and fails to account for how waste treatment influences stakeholder’s decision-making. Given the complexity of C&D waste management, which involves multiple interdependent procedures, managers often face difficulty identifying the most efficient and effective solutions^5^. Moreover, BIM-based management frameworks are constrained by BIM’s technological limitations^23^. This approach enables only a preliminary estimation of waste quantification during the planning stage without the ability to make precise, real-time predictions based on on-site implementation and disassembly. While theoretical models and individual technological applications of BIM and DT have been widely explored, there remains a pressing need for a more comprehensive, multi-stage demolition planning methodology.

Additionally, although previous studies have acknowledged the financial benefits of C&D waste recycling, few have proposed detailed, structured strategies for planned demolition and waste management tailored to specific buildings. To address this research gap, this study aims to integrate BIM and DT technologies into an enhanced conceptual framework for C&D waste management. By leveraging this framework, managers can simulate the demolition process in advance, mitigating potential risks and optimizing decision-making. Furthermore, it enables a deeper understanding of building conditions before demolition, facilitating more efficient and sustainable waste management practices.

## Methods

This study employs a hybrid research approach to develop a comprehensive system framework for C&D waste management. The methodology integrates data collection, building demolition analysis, and economic benefit evaluation.

**Fig. 1:**
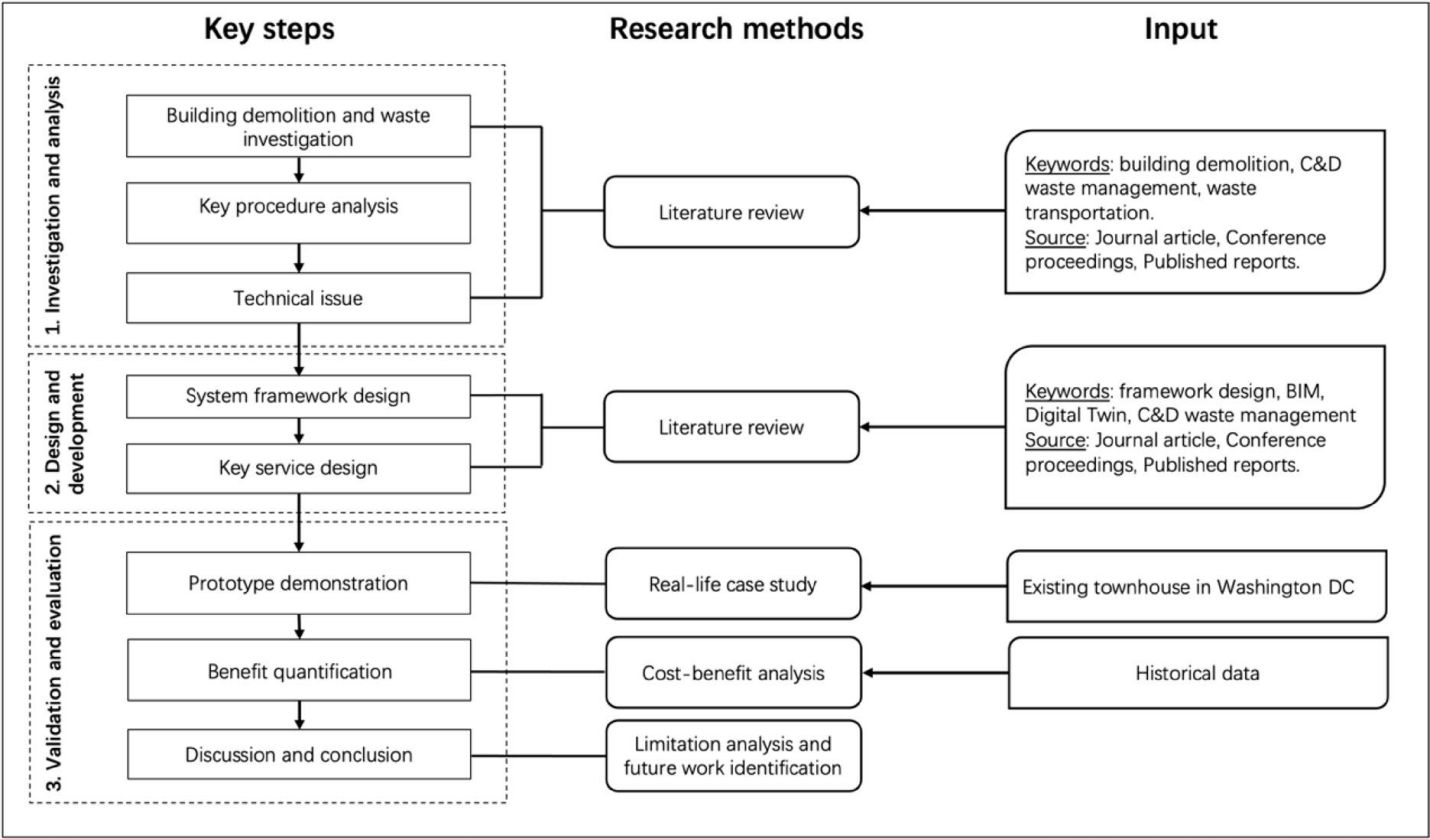
Methodology.

As illustrated in Fig. 1, the research process is structured into three key stages:

- *Analysis of Existing C&D Waste Management Frameworks* The first stage involves critically examining the key processes within current C&D waste management frameworks. This analysis identifies inefficiencies and areas for optimisation and improvement.
- *Development of a Digital Twin-Based System Framework* In the second stage, Digital Twin technology is utilised to construct an enhanced system framework for the entire C&D waste management process. This framework facilitates real-time data integration, improves decision-making, and enhances efficiency.
- *Simulation and Economic Benefit Analysis* The final stage involves simulating the entire waste management process using BIM-Dynamo. This simulation enables analysis of the correlation between waste recovery rates and financial benefits. The results provide insights into the economic viability of different waste management strategies.

### Implementation of BIM-Navisworks for sustainability building demolition

The previous research focused on the reconstruction of an existing townhouse in Washington, D.C., with the goal of achieving net-zero energy performance. Kaewunruen et al.^32^ proposed reconstruction design that minimised energy consumption by incorporating renewable energy technologies and energy-efficient construction practices. Building upon previous research, the current study shifts its focus to the end-of-life phase of the building, specifically investigating how the existing townhouse can be leveraged to optimise economic benefits through improved construction and C&D waste management practices. The aim is to enhance sustainability outcomes by integrating material recovery strategies, such as reuse and recycling, into the demolition process. The C&D management framework developed in this study was tailored to the specific characteristics of the case study; however, it is designed to be adaptable and scalable to other demolition projects with appropriate customisation. This adaptability allows for broader application of the proposed workflow in diverse contexts, supporting more sustainable and economically viable demolition practices.

**Fig. 2:**
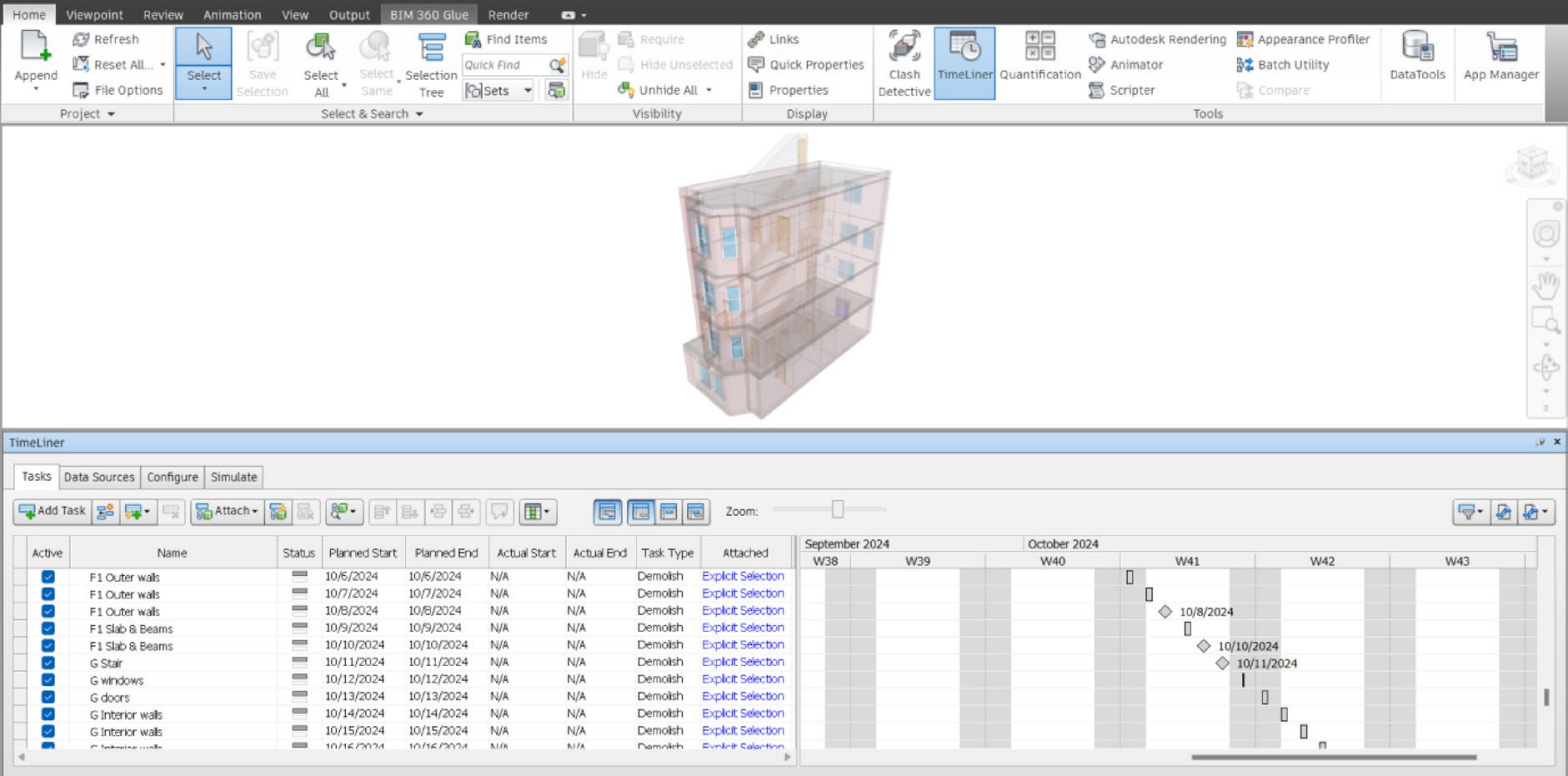
The implementation of BIM-Navisworks for the existing townhouse in Washington, D.C.

With the increasing emphasis on green building development, it is crucial to implement a structured demolition plan rather than resorting to the direct destruction of existing structures. A planned demolition approach can reduce the environmental impact of disassembly waste by at least 50%^33^. The BIM-Navisworks software plays a pivotal role in optimising demolition planning. By simulating the demolition process through animation, BIM-Navisworks enhances visualization, making the disassembly process more intuitive and displaying critical building information. Additionally, by integrating 4D modelling, the demolition plan can be effectively linked with the construction timeline, allowing for better schedule coordination and resource allocation^34^. Figure 2 illustrates the application of BIM-Navisworks in building demolition planning for the case study, highlighting its role in optimising demolition processes and decision-making.

The process of using BIM-Navisworks for demolition planning involves several key steps:

1. *Importing the Revit Model* The initial step involves importing the BIM-based Revit model into BIM-Navisworks to facilitate a structured disassembly process.
2. *Verification of Component Information* Ensuring all relevant building components are correctly identified and categorised for demolition.
3. *Creation of Disassembly Views* Generating specific views within BIM-Navisworks to represent different stages of the disassembly process visually.
4. *Construction Schedule Formulation* Defining the demolition sequence for each building component ensures an organized and efficient execution plan.
5. *Development of Disassembly Animation* Simulating the demolition process through BIM-Navisworks animations provides stakeholders with a clear and detailed step-by-step visualisation of the planned demolition.

Furthermore, BIM-Navisworks enables collision detection, allowing potential risks and conflicts in the demolition plan to be identified and resolved before execution. This proactive approach enhances safety, reduces errors, and improves overall demolition efficiency^34^. By leveraging BIM-driven planning, construction professionals can achieve more sustainable, cost-effective, and environmentally friendly demolition processes.

### Enhanced C&D waste management framework utilising DT technology

**Fig. 3:**
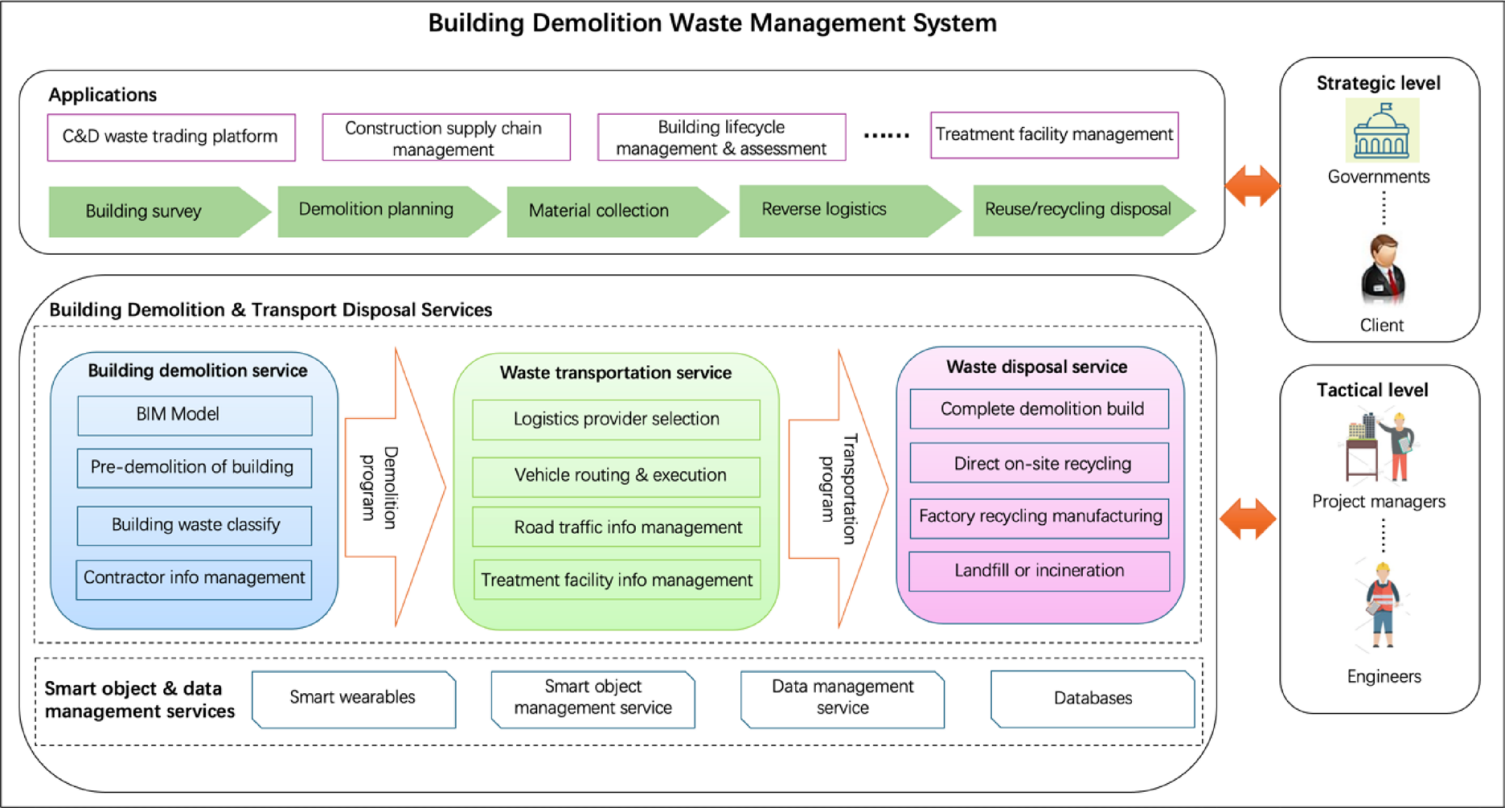
C&D Waste Management System.

This study builds upon the C&D waste management framework proposed by Yeheyis et al.^22^, which is based on the life cycle of buildings. A more comprehensive system framework is developed to optimise management practices, incorporating key elements such as demolition planning, transportation logistics, post-demolition treatment strategies, and stakeholder engagement, as illustrated in Fig. 3.

The framework is structured into multiple layers, each serving a distinct function:

- *Application Layer* This layer integrates Digital Twin technology with demolition waste management. By utilising design-driven applications, Digital Twin enables real-time monitoring and control, improving the efficiency of waste tracking, resource allocation, and decision-making.
- *Data Management and Transportation Services Layer* This component facilitates intelligent data acquisition and management through Internet of Things (IoT) devices, including wearable smart devices, scanning technologies, and mobile platforms. These devices capture and store real-time data in an intelligent database, allowing stakeholders and managers to efficiently monitor, analyse, and control demolition waste activities^35^.

This system framework enhances waste traceability, improves operational efficiency, and supports sustainable demolition practices by leveraging digital twin and IoT technologies. It provides a data-driven approach to C&D waste management, ensuring better stakeholder coordination while minimising environmental impacts.

### Optimised transportation framework for C&D management

**Fig. 4:**
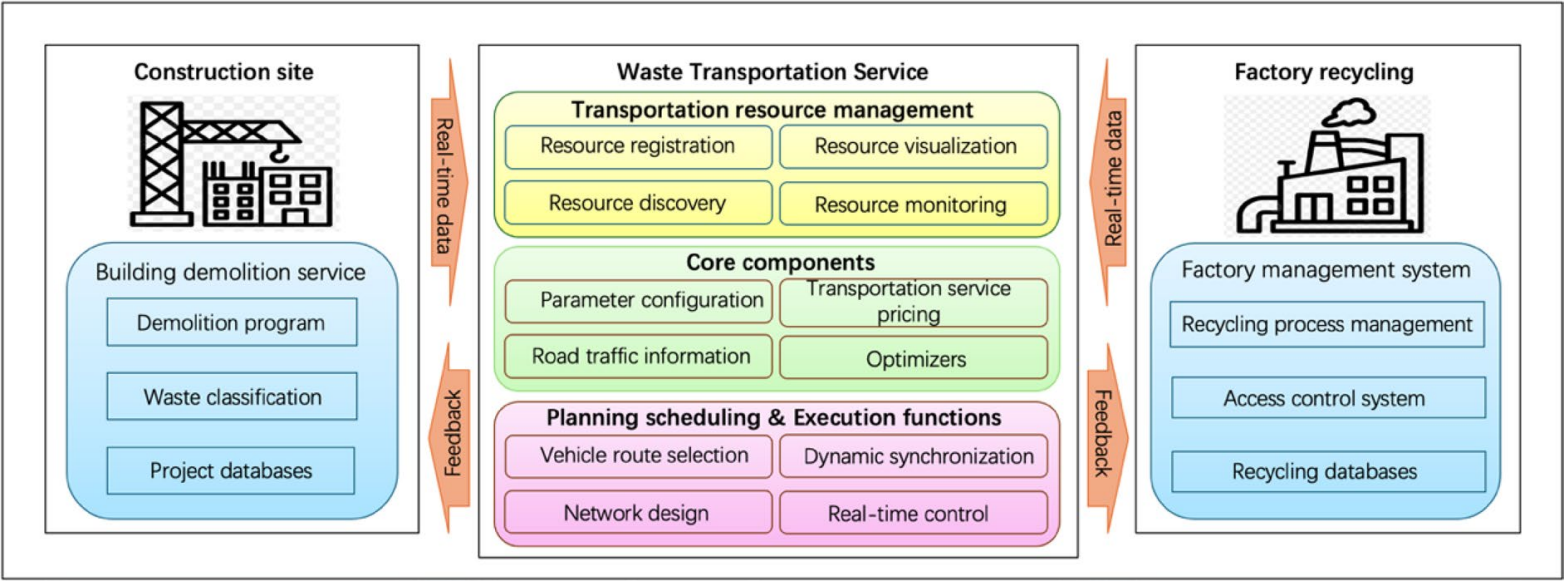
Waste Transportation Service.

Figure 4 presents the transportation flowchart for moving demolition waste from the demolition site to the corresponding recycling or treatment facility. This study adopts a systematic classification approach based on waste type and size, ensuring efficient sorting and disposal processes. To enhance data accuracy and tracking, workers are equipped with wearable intelligent devices that capture and store real-time data, which is then uploaded to a centralised project database. The transportation management system consists of three core modules:

- *Transportation Resource Management* This module optimises the allocation and utilisation of transportation resources, ensuring efficient vehicle routing and load balancing.
- *Core Composition*,* Planning*,* and Scheduling* This involves strategically organising waste transportation, including route planning, scheduling, and load distribution to minimise fuel consumption and environmental impact.
- *Execution and Real-Time Data Transmission* This module facilitates live data sharing between construction sites and recycling plants, enabling real-time feedback loops to optimise logistics operations^36^.

To address the challenges associated with waste transportation in C&D waste management, Bao et al.^37^ developed an Internet-based framework that records all transportation details and enables real-time tracking. This framework enhances operational transparency, improves logistical efficiency, and ensures timely waste processing, contributing to sustainable construction waste management practices. In this study, the recycling process of waste transportation services is carried out by referencing the Internet-based framework employed to collect and monitor data throughout the transportation process.

### Financial-Benefit analysis of waste management

This study proposes a conceptual framework for managing C&D waste based on three primary waste disposal methods:

- *Direct On-Site Sale and Recycling* Salvageable materials are immediately resold or reused at the demolition site, minimising waste generation and transportation costs.
- *Indirect Recycling* Waste materials are transported to recycling facilities for further processing and repurposing.
- *Landfilling* Non-recyclable materials are disposed of in designated landfills, although this is considered the least sustainable option.

The final output of this study will be each method’s recycling proportions and assess the financial benefits associated with different recycling levels. This aims to theoretically validate the feasibility of the proposed C&D waste management framework and provide strategic insights for enhancing waste management practices. Due to material loss and volume fluctuations during demolition, the waste volume derived from the BIM model cannot be directly used for cost-benefit calculations. This study introduces a waste volume change factor $\:{C}_{y}$ to improve accuracy and reflect real-world conditions^38^. The volume change factor established by the Andalusian Construction Cost Database (BCCA)^39^ will differ from the parameters outlined in the governmental construction guidelines of other nations.

Table 1 presents the volume change factors for various materials (e.g., concrete, steel, glass, and wood), and the final waste volume is calculated using the following equation:

1 $$\:{V}_{F}={V}_{O}\times\:{C}_{y}$$

Where:

- $\:{V}_{F}$​ represents the final volume of a material post-demolition.
- $\:{V}_{O}$ denotes the estimated material volume based on BIM-simulated demolition.
- $\:{C}_{y}$​ is the waste volume change factor, accounting for material loss, contamination, and other influencing factors as defined by BCCA^39^.

By integrating BIM-based modelling with real-world adjustment factors, this framework enhances the accuracy of demolition waste predictions, ultimately optimising resource recovery and financial planning in C&D waste management.

**Table 1:** Waste volume change Factor.

Material	Volume change factor:$\:{C}_{y}$
Concrete	1.10
Steel	1.30
Glass	1.05
Wood	1.05

By the “3R” principle (Reduce, Reuse, Recycle)^40^, this study formulates a waste treatment strategy based on the type and composition of dismantled materials as defined in the BIM model. To evaluate the economic efficiency of the proposed C&D waste management framework, the financial-benefit analysis is conducted using the following Eq^41^, where the net income is calculated as:

2 $$\:C={C}_{P}+{C}_{R}+{(-C}_{C})+\left(-{C}_{L}\right)+(-{C}_{D})$$

- $\:C$ is the total profit of the disposal scheme.
- $\:{C}_{C}$ is the on-site collection cost.
- $\:{C}_{P}$​ is the direct sale cost.
- $\:{C}_{R}$ is the recovery cost at the treatment centre.
- $\:{C}_{L}$​ is the landfilling cost.
- $\:{C}_{D}$​ is the total transportation cost.

The proportion of waste allocated to each disposal method satisfies the following constraint:

3 $$\:1={X}_{P}+{X}_{R}+{X}_{L}$$

where:

- $\:{X}_{P}$, $\:{X}_{R}$ and $\:{X}_{L}$​ represent the proportions of waste designated for on-site recycling and sale, factory recycling and landfilling respectively.

The financial components are further defined as follows:

4 $$\:{C}_{C}={Q}_{i}\times\:{P}_{i}$$

5 $$\:{C}_{P}={Q}_{i}\times\:{X}_{P}\times\:{R}_{i}$$

6 $$\:{C}_{D}={Q}_{i}\times\:{(X}_{R}+{X}_{L})\times\:{D}_{i}\times\:{T}_{i}$$

7 $$\:{C}_{R}={Q}_{i}\times\:{X}_{R}\times\:{R}_{i}$$

8 $$\:{C}_{L}={Q}_{i}\times\:{X}_{L}\times\:{L}_{i}$$

where:

- $\:{Q}_{i}$ is the quantity of waste category iii.
- $\:{P}_{i}$, $\:{R}_{i}$, $\:{L}_{i}$and $\:{T}_{i}$ represent Unit prices for on-site collection, recycling, landfilling, and transportation, respectively.
- $\:{D}_{i}$ denotes transportation distance.

For this study, it is assumed that no material loss occurs throughout the entire waste disposal process and that the recovery rate and landfill rate are set to 1 (i.e., 100% recovery and 100% landfill disposal, respectively). These assumptions provide a baseline model, which can later be adjusted to reflect real-world inefficiencies, such as material loss, contamination, and processing costs. Integrating BIM-driven waste quantification with financial modelling aims to provide a comprehensive cost-benefit analysis for sustainable demolition waste management, ultimately supporting informed decision-making in C&D waste recovery and disposal planning.

## Results

This section focuses on the methodology for simulating the C&D waste management process outlined in the proposed conceptual framework. To evaluate its effectiveness, the framework is applied to an existing townhouse in the Washington, D.C., area. The study assesses the financial benefits of implementing BIM and Digital Twin technologies in demolition waste management by comprehensively analysing cost savings and resource optimisation. For this simulation, architectural and structural data of existing townhouses were obtained from the study by Kaewunruen et al.^32^. This data is the foundation for accurately modelling the demolition and waste management process, ensuring that the simulation reflects real-world scenarios. By integrating BIM and Digital Twin technologies, the study aims to enhance demolition planning, optimise waste classification and transportation, and quantify the economic and environmental benefits of improved C&D waste management practices.

### Demolition waste management and BIM-integrated planning

**Fig. 5:**
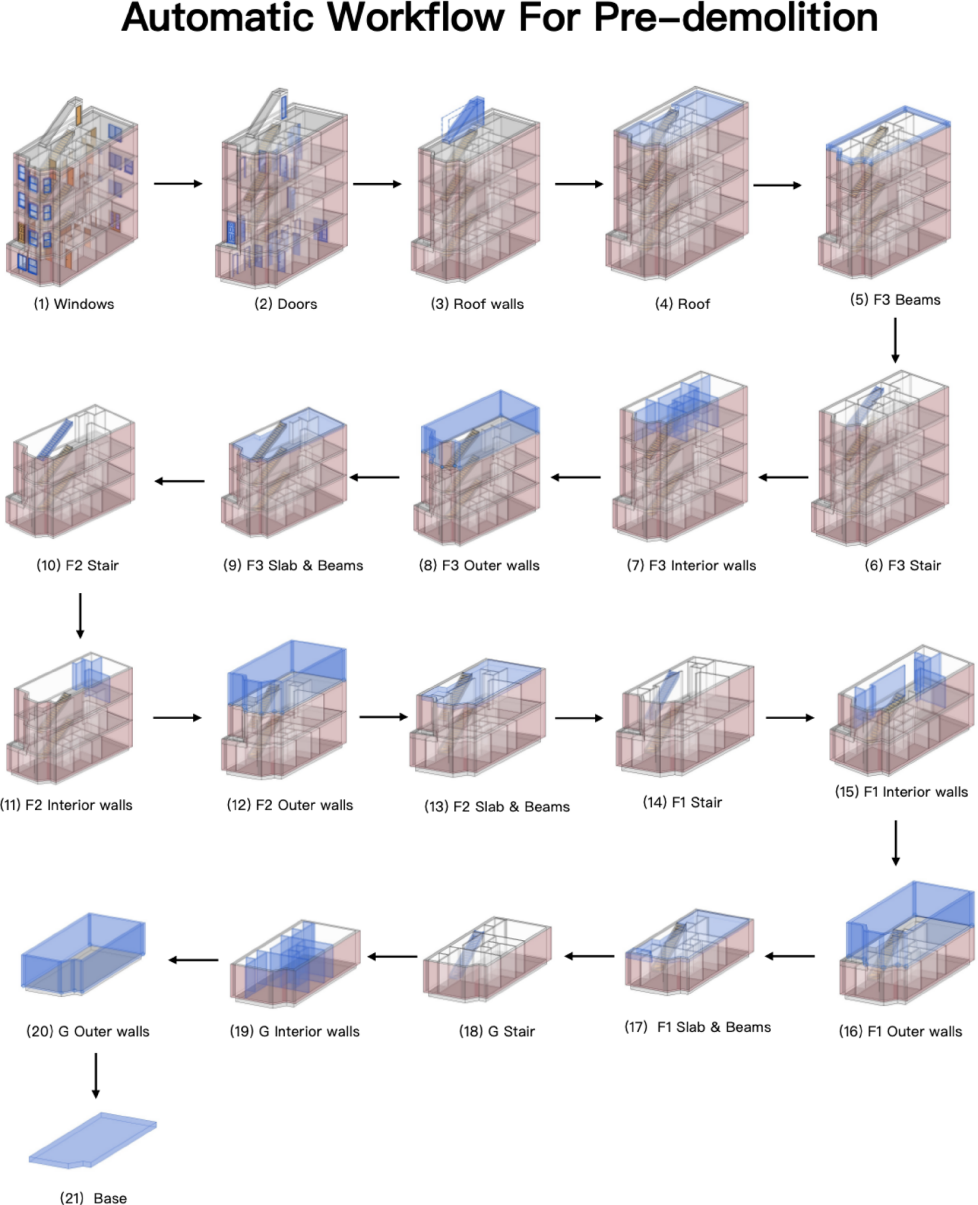
The Demolition Process of Existing Townhouse in Washington, D.C.

Effective demolition waste management necessitates the development of a comprehensive demolition plan, including a three-dimensional (3D) model of the existing building. In this study, the 3D model of a townhouse in the Washington, D.C. area is first constructed and then imported into BIM-Navisworks to facilitate precise planning and execution. The process begins with classifying BIM object categories and identifying relevant attributes, such as the dismantling method and the corresponding recycling approach. This classification ensures that each building component is appropriately managed during demolition, optimising waste separation and material reuse. The next step involves establishing a systematic demolition sequence. In alignment with standard demolition practices, the deconstruction process follows a top-down approach, reversing the original construction order to maintain structural stability and ensure safety. Particular attention is given to the disassembly of prefabricated components, where beams and slabs are removed as integrated units, while walls and columns are dismantled collectively. Figure 5 presents the fundamental demolition sequence, illustrating the phased deconstruction process in conjunction with the demolition schedule developed in Navisworks. The demolition plan for the case study commences with removing the building’s windows, followed sequentially by the doors and roof walls. Upon completion of roof removal at Stage 4, the subsequent phases involve systematically dismantling the beams, staircases, and interior and exterior walls, proceeding progressively from the third floor down to the ground floor. The demolition workflow is monitored and updated in real time, utilising an imported demolition plan from a spreadsheet to guide and track progress.

To highlight, the 3D model of the existing townhouse located in Washington, D.C., including static structural elements such as beams, columns, and walls, is developed using Autodesk Revit. This BIM model was subsequently imported into Navisworks to support detailed demolition planning. Within the Navisworks environment, the BIM model was integrated with a predefined demolition schedule to quantify the building components designated for removal at each stage of the demolition process. Upon finalisation of the demolition plan, the model was further integrated with three distinct waste management strategies. These strategies encompass alternative treatments for materials resulting from demolition, including reuse, recycling, and landfilling. This integration enabled a comparative analysis of the financial implications associated with each waste management approach. The automation of this workflow was achieved through the use of Dynamo scripts, which allowed for real-time parametric linking between the demolition schedule, component classification, and corresponding waste management strategy. This facilitated dynamic visualisation and monitoring of component removal and material allocation throughout the demolition timeline, thereby supporting informed decision-making within a C&D waste management framework.

While the outcomes presented are specific to the case study, the workflow architecture is inherently modular and adaptable. Its application to other demolition projects is feasible with appropriate customisation to reflect project-specific parameters, data formats, and model structures. A key limitation, however, lies in its reliance on structured and consistently formatted data, which may require additional preprocessing for broader implementation. A detailed discussion of the financial benefits and implementation outcomes is provided in the subsequent sections.

In this study, the primary categories of C&D waste include concrete, metal, wood, and glass. According to existing research, a significant proportion, ranging from approximately 50–95% of construction and C&D waste can be effectively recycled or reused. This potential is primarily determined by the material properties, with both inert materials (e.g., concrete, sand) and non-inert materials (e.g., glass, wood, plastic) offering opportunities for recovery and diversion from landfill^42^. Based on these findings, the present study proposes three distinct waste treatment strategies aimed at optimising the management of C&D waste. Each strategy differs in terms of recycling efficiency and reliance on landfill disposal. Importantly, the proposed waste management plans are designed to satisfy the constraints outlined in Eq. (3), ensuring both environmental and economic feasibility within the demolition framework.

- **Plan A**: 50% of C&D waste is recycled, with 10% on-site recycling & sale (X_p_) and 40% transported to recycling facilities (X_R_). The remaining 50% is disposed of in landfills (X_L_).
- **Plan B**: 80% of C&D waste is recycled, including 10% for on-site recycling & sale and 70% processed at recycling facilities. The remaining 20% is landfilled.
- **Plan C**: 95% of C&D waste is recycled, with 10% on-site recycling & sale and 85% transported to recycling facilities. Only 5% is sent to landfills.

These waste management plans provide a structured approach to balancing economic and environmental sustainability, offering varying levels of material recovery while minimising landfill disposal. The selection of an optimal strategy depends on project-specific constraints, financial considerations, and regulatory requirements.

### Waste dismantling and transportation strategy

The quantity of dismantled waste is presented in Table 2. Utilising BIM-Navisworks for pre-demolition planning, this study assumes that 10% of dismantled components can be directly reused and sold on-site. In practice, the recycling of reinforced concrete structures is primarily achieved through crushing and separation into aggregate and steel. Since wood is a natural material, it can be repurposed into engineered wood products^43^. Current research indicates that the predominant treatment method for dismantled glass involves reprocessing, with some materials being converted into concrete aggregates^44^. A study found that the average transport distance of C&D waste by truck ranges from 10 to 30 kilometres^45^. This study references these existing waste management solutions and assumes that the transportation distance from the demolition site to the treatment facility is approximately 10 km. The transportation plan is managed through a real-time data platform integrated with a DT system. When the accumulated waste reaches a predefined transportation threshold, an automated “Transport Order” is generated. The total mass of material was calculated using Eq. 9, with the material density referenced from Ansys Granta EduPack 2021^46^.

9 $$\:{Mass\:(Q}_{t})={Volume\:(V}_{F})\times\:Density\:\left({\uprho\:}\right)$$

Within the “Transportation Task Allocation” framework, the data platform assigns tasks to specific drivers via smartphone-based communication. Simultaneously, drivers provide real-time feedback regarding waste transportation and recycling status through mobile terminals, ensuring an efficient, closed-loop system for waste transportation management. This dynamic approach enhances logistical efficiency while reducing environmental and operational costs.

**Table 2:** Quantity of demolished Waste.

Material	Area (m^2^)	Original volume: V_0_(m^3^)	Volume change factor (C_y_)	Final volume: V_F_ (m^3^)	Density: ρ (t/m^3^)	Total mass: Q(t)
Concrete	–	292.78	1.10	322.06	2.40	772.95
Steel	–	81.75	1.30	106.28	7.85	834.26
Glass	29.45	0.15	1.05	0.15	2.20	0.34
Wood	44.60	1.81	1.05	1.90	0.80	1.52

### Analysis of financial benefits

**Fig. 6:**
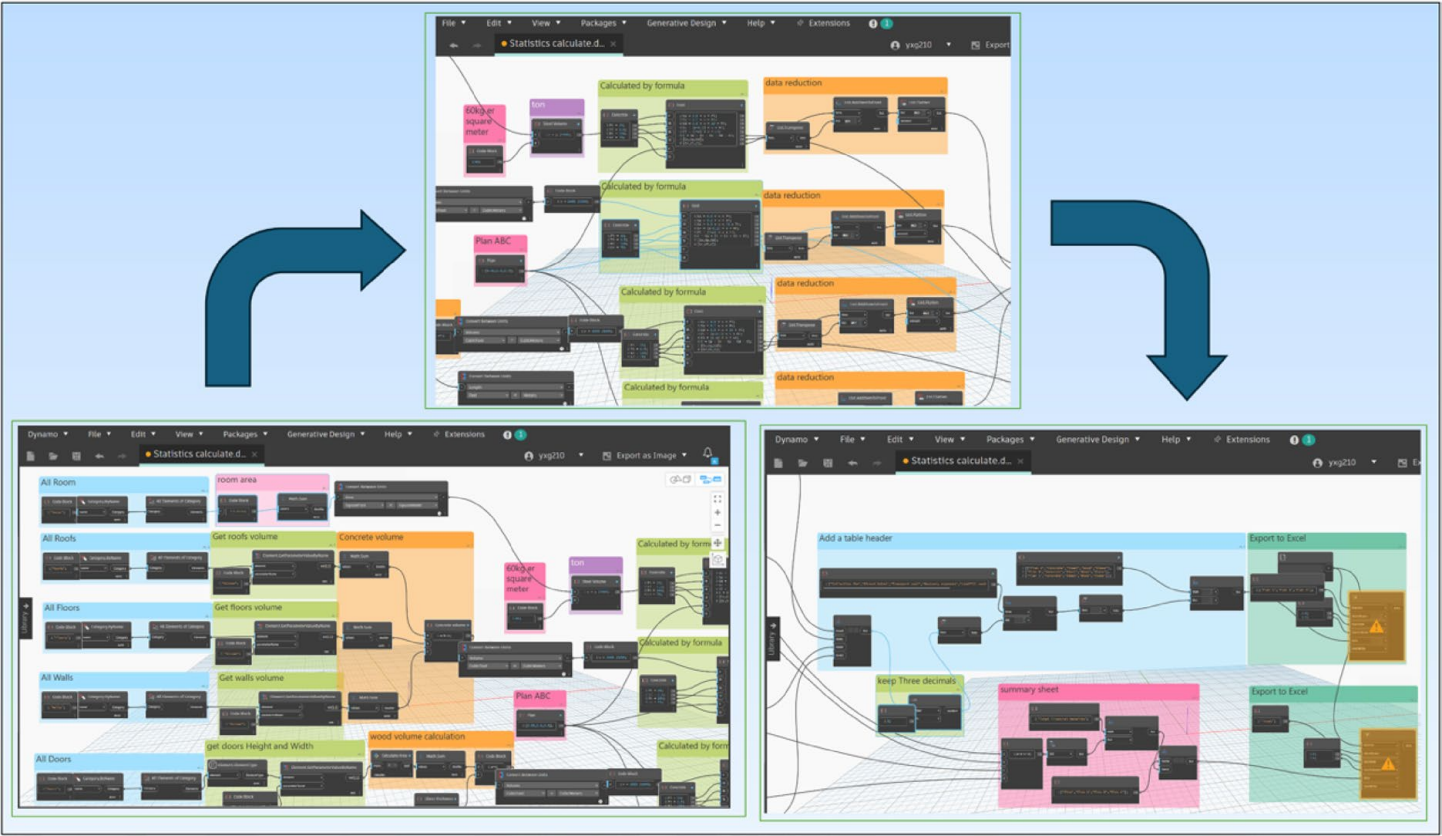
Process of BIM-Dynamo.

Table 3 provides a comprehensive breakdown of unit costs associated with the treatment of various categories of C&D waste in the Washington, D.C., area. These costs encompass multiple components, including waste collection fees, transportation expenditures, material recovery processing costs, and landfill disposal charges. The data reflects region-specific economic factors and operational practices, offering valuable insight into the financial implications of C&D waste management strategies and supporting cost-benefit analyses for sustainable demolition planning. The data is sourced from the Metropolitan Washington Council of Governments and supplemented with information from local organizations, including American Recycler, the Institute for Local Self-Reliance, and Zero Waste DC. Furthermore, by utilising BIM-Dynamo software enables precise quantification of the total demolition volume of the existing building model, facilitating an in-depth analysis of the financial benefits associated with different waste management strategies. Figure 6 illustrates the sequential process, beginning with the initial static calculation and progressing to the real-time, automated demolition workflow executed using Dynamo, based on the parameters defined in this study. The system architecture is designed to be adaptable to varying demolition plans, allowing for immediate updates and dynamic reconfiguration within the model. In this case study, the system processes input data derived from the three proposed demolition scenarios, Plans A, B, and C, as described in previous sections, allowing for a comparative analysis of material flows and resource recovery potential under each approach. Tables 3 and 4, and 5 present the detailed cost analyses corresponding to the three proposed C&D waste treatment plans. Each table outlines the breakdown of waste treatment fees for various material categories, including concrete, steel, wood, and glass. These calculations encompass associated costs such as collection, transportation, material recovery, and landfill disposal. In addition to the treatment expenses, the tables also account for the potential financial returns generated through the reclamation and resale of reusable materials. The resulting net financial benefits are computed for each plan, thereby enabling a comparative assessment of the economic efficiency and resource recovery potential across the different demolition strategies.

As illustrated in Table 6, increasing the recycling rate of C&D waste from 50 to 80% and 95% yields a substantial improvement in financial returns. The analysis demonstrates that, at a 50% recycling rate, the process results in a financial deficit, with a negative net benefit of -£10,627.62. This indicates that under low recycling conditions, the costs associated with collection, transportation, and treatment outweigh the economic value of recovered materials. However, when the recycling and reuse rate is increased to 80%, the financial outlook shifts positively, resulting in a net benefit of £70,418.51. Further improvement is observed at a 95% recycling rate, where the net monetary benefit reaches £110,941.57, as presented in Table 7. These findings underscore the strong correlation between higher recycling efficiency and economic viability, highlighting the importance of maximizing material recovery rates in sustainable demolition practices. Therefore, based on the simulation calculations conducted through the BIM-Dynamo integration, the results indicate that the intelligent waste management approach proposed in this study’s conceptual framework has the potential to generate positive economic returns in the context of building demolition and C&D waste management. The analysis reveals a strong positive correlation between the material recycling rate and the overall financial benefits, suggesting that higher levels of material recovery significantly enhance the cost-effectiveness of demolition activities. This correlation highlights the economic viability of adopting a BIM-driven decision-support system, which enables dynamic modelling, real-time scenario analysis, and optimisation of resource recovery strategies. As such, the proposed approach not only supports environmentally sustainable practices but also offers measurable financial advantages, reinforcing the value of digital technologies in circular economy applications within the construction sector.

**Table 3:** Material-Specific cost Analysis.

	Collection fees (£/t)$\:{(P}_{i})$	Transport cost (£/t/km)$\:\left({T}_{i}\right)$	Recovery expenses (£/t)$\:\left({R}_{i}\right)$	Landfill cost (£/t)$\:\left({L}_{i}\right)$
Concrete	15.28	1.91	114.60	57.30
Steel	16.81	1.91	106.96	57.30
Glass	19.10	1.91	99.32	53.48
Wood	15.28	1.91	84.04	45.84

**Table 4:** Financial-Benefit of plan A.

Plan A	Concrete	Steel	Wood	Glass
Collection fees $\:{(C}_{c})$	−11,810.63	−14,023.89	−23.17	−6.49
Direct sales $\:{(C}_{p})$	8,857.97	8,923.23	12.74	3.37
Transport cost $\:{(C}_{D})$	−13,286.96	−14,340.91	−26.06	−5.84
Recovery expenses $\:{(C}_{R})$	35,431.90	35,692.93	50.97	13.49
Landfill cost $\:{(C}_{L})$	−22,144.93	−23,901.51	−34.75	−9.08
Net financial benefits	−2,952.66	−7,650.15	−20.27	−4.54

**Table 5:** Financial-Benefit of plan B.

Plan B	Concrete	Steel	Wood	Glass
Collection fees $\:{(C}_{c})$	−11,810.63	−14,023.89	−23.17	−6.49
Direct sales $\:{(C}_{p})$	8,857.79	8,923.23	12.74	3.37
Transport cost $\:{(C}_{D})$	−13,286.96	−14,340.91	−26.06	−5.84
Recovery expenses $\:{(C}_{R})$	62,005.82	62,462.62	89.20	23.61
Landfill cost $\:{(C}_{L})$	−8,857.97	−9,560.61	−13.90	−3.63
Net financial benefits	36,908.22	33,460.45	38.81	11.03

**Table 6:** Financial-Benefit of plan C.

Plan C	Concrete	Steel	Wood	Glass
Collection fees $\:{(C}_{c})$	−11,810.63	−14,023.89	−23.17	−6.49
Direct sales $\:{(C}_{p})$	8,857.97	8,923.23	12.74	3.37
Transport cost $\:{(C}_{D})$	−13,286.96	−14,340.91	−26.06	−5.84
Recovery expenses $\:{(C}_{R})$	75,292.78	75,847.47	108.31	28.67
Landfill cost $\:{(C}_{L})$	−2,214.49	−2,390.15	−3.48	−0.91
Net financial benefits	56,838.67	54,015.75	68.34	18.81

**Table 7:** Comparison of total Financial-Benefit among three Plans.

Plan	Plan A	Plan B	Plan C
Net financial benefits	−10,627.62	70,418.51	110.941.57

## Discussion and limitations

The first key issue addressed in this study is the optimisation of BIM and DT technologies for C&D waste management. The proposed conceptual framework is grounded in a BIM-driven Digital Twin approach, wherein the demolition process of the target building is simulated for advanced planning. Real-time data transmission ensures continuous updates to the Digital Twin system, enabling managers to make informed decisions throughout the demolition and waste management process. Additionally, financial-benefit assessments of waste recovery are conducted using BIM-Dynamo, which facilitates the practical application of BIM-driven Digital Twin technology in C&D waste management. BIM-Dynamo is critical for demonstrating how BIM-driven DT technologies can enhance waste management processes. In the case study, the BIM model of the existing house is integrated with the proposed method for estimating waste processes in real-time.

The second research question pertains to the critical factors influencing the development of an optimal waste management plan. In this study, waste management planning primarily involves classifying and treating different waste types, including landfill disposal, direct sale, recycling, and material reprocessing. The primary factors include environmental impact, economic benefits, and overall feasibility. However, while this study focuses on the most commonly recognised economic advantages, it does not account for additional cost variables such as labour, time constraints, and equipment leasing. In practical demolition projects, these factors significantly determine whether to adopt controlled demolition strategies or conventional blasting methods. The primary objective of comparing the three recycling plans is to evaluate their relative environmental and economic performance under consistent assumptions. A uniform recycling rate is applied across all waste materials to ensure a fair and controlled comparison. This approach mitigates variability introduced by material-specific factors, allowing for a direct assessment of the differences between the recycling strategies rather than disparities in material recyclability. A standardised recycling rate establishes a consistent baseline, aligning with industry benchmarking practices and simplifying the analytical process. While material-specific rates could offer finer granularity, they may introduce unnecessary complexity, particularly in cases with limited localised or project-specific data. This study ensures transparent and robust comparisons across the three recycling plans by prioritising methodological consistency. Future research could explore the impact of material-specific recycling rates on C&D waste management and how BIM-driven DT could support dynamic adjustments for varying material recovery rates.

The third research objective is to identify strategies for maximising economic benefits while promoting environmental sustainability in C&D waste management. A key approach involves meticulous sorting and classification of demolition waste to enhance material recovery rates and ensure higher-quality recycling. By categorising waste materials appropriately, reusable components can either be resold or repurposed for future construction projects, generating additional revenue. Furthermore, optimising the demolition plan by selecting environmentally friendly dismantling methods, rather than conventional demolition by blasting, further contributes to sustainability objectives. In addition, long-term monitoring of C&D management development and frequent data updates are essential for enhancing the digital model and effectively demonstrating the benefits of the physical model in real-world applications. This study focuses on implementing a BIM-driven Digital Twin to identify and evaluate various recycling plans aimed at enhancing financial benefits during the demolition phase. Future research should extend this work by incorporating a more comprehensive assessment of environmental and operational costs to improve overall sustainability and more effectively minimise environmental impacts.

However, certain limitations in this research should be addressed in future studies. In this scenario, the transportation distance from the demolition site to the treatment facility was assumed to be 10 km; however, this distance may vary depending on project locations and vehicle sizes^45^. The BIM-driven model proposed in this research can support such adjustments in future applications. Furthermore, the financial benefits and environmental impacts of the C&D management plan were evaluated solely based on the treatment of materials during the demolition phase. Additional factors, such as energy consumption during demolition, transportation energy^45^, waste recycling processes involving different material compositions^47^, and labour costs, are expected to influence the financial analysis of C&D management and further enrich the sensitivity analysis of financial benefits. Moreover, the feasibility of recycling can be varied depending on market conditions and governmental regulations^40^. These specific aspects should be incorporated into future research to enable a more comprehensive evaluation of C&D management practices. On the other hand, the research presented here focuses on analysing the financial benefits of three different recycling plans in C&D management during the demolition phase.

During the demolition phase, Carbon dioxide (CO₂) emissions should be systematically integrated into C&D waste management strategies. These emissions arise from various sources, including energy consumption during demolition activities, transportation of waste, the processing remanufacturing of recyclable materials, landfilling, etc^48^. A comprehensive assessment of these greenhouse gas contributions is essential for accurately evaluating the environmental impact of demolition operations. By incorporating CO₂ emissions into the C&D management framework, stakeholders can identify more sustainable practices and promote informed decision-making. This additional consideration can significantly enhance the environmental sustainability of the built environment, particularly in efforts to reduce the carbon footprint associated with end-of-life building processes.

Future research could expand these scopes by comparing the costs associated with recycling processes and using recycled materials in new construction against new materials, thereby demonstrating the environmental and economic advantages of component recycling.

## Conclusion and future prospects

This study aims to advance the adoption of BIM and DT technology in building demolition and waste management. By refining the existing demolition waste management framework, the research aims to optimise waste handling and planning following the demolition of ageing structures. The proposed framework establishes a dynamic DT environment by integrating BIM with real-time data processing. While auxiliary technologies like wearable scanners and mobile applications provide supplementary data channels, the system’s core intelligence resides in the bidirectional synchronisation between the BIM model and operational data streams. This BIM-DT integration enables (1) continuous model updating through embedded IoT data pipelines, (2) automated generation of transmission instructions via built-in logic modules, and (3) closed-loop coordination of physical and digital project states. The framework prioritises BIM-to-DT data flows as the primary mechanism for maintaining model fidelity and supporting decision-making processes.

Implementing BIM-Navisworks enables pre-demolition simulations, helping identify potential risks and optimise demolition strategies in advance. Additionally, real-time data transmission ensures project managers access up-to-date information, enhancing decision-making during demolition operations. This study employs simulation-based verification to validate the effectiveness of the proposed conceptual framework for C&D waste management. BIM-Dynamo is utilised to assess the financial feasibility of waste recycling, demonstrating that a higher recycling rate significantly enhances economic returns. The findings indicate that integrating BIM and DT technologies in demolition waste management improves efficiency and promotes sustainable demolition practices by maximising material recovery.

While introducing the conceptual framework for the comprehensive management of C&D waste demonstrates the potential to enhance demolition waste management, several limitations must be addressed. Key challenges include the accuracy of target building modelling, long transportation distances for waste disposal, data integrity issues, and the effectiveness of managerial decision-making. The framework should be further refined to achieve more sustainable construction practices based on regional variations in recycling preferences and transportation logistics.

To advance the effectiveness of C&D waste management, several areas warrant further investigation:

- *Integration of Artificial Intelligence (AI) and Intelligent Systems* Future research should explore incorporating AI and other advanced technologies to enhance decision-making, minimise risks, and improve efficiency in the waste management process. AI-driven predictive analytics could optimise waste sorting, transportation routes, and material reuse strategies.
- *Enhanced Building Modelling Techniques* A significant challenge in demolition projects is the lack of detailed as-built documentation for older buildings. Future studies should consider employing drones, LiDAR scanning, and other advanced imaging technologies to generate accurate 3D models for better planning and resource estimation.
- *Regionalised Financial Analysis of Waste Recycling* Given the variability in recycling infrastructure and policies across different regions, future research should analyse financial benefits based on localised recycling rates. A region-specific approach would optimise waste management strategies while maximising economic returns and environmental sustainability.
- *Sensitivity Analysis of Net Financial Benefits* The economic feasibility of the recycling process may vary significantly depending on market conditions and governmental regulations. Future research should incorporate a broader range of cost scenarios to enhance the robustness and applicability of the findings across diverse geographical contexts. These scenarios may include transportation distance and vehicle type variations, labour costs, energy consumption, and associated carbon emissions. A more comprehensive sensitivity analysis considering these factors would contribute to a deeper understanding of the financial implications and support more context-specific decision-making in C&D waste management.

By addressing these research gaps, future studies can refine the proposed framework further, ensuring that demolition waste management is not only more efficient but also aligned with sustainable construction principles.

## Data Availability

The data that support the findings of this study are available from the corresponding author upon reasonable request.
